# Hemoglobin Concentrations and Prevalence of Anemia During Pregnancy: Results from the Brazilian Maternal and Child Nutrition Consortium

**DOI:** 10.1016/j.cdnut.2025.107458

**Published:** 2025-05-08

**Authors:** Nathalia Cristina de Freitas-Costa, Thais Rangel Bousquet Carrilho, Paula Normando da Costa, Helena M Constante, Elizabeth Fujimori, Ana Paula Sayuri Sato, Gilberto Kac, Brazilian Maternal and Child Nutrition Consortium, Adauto Emmerich Oliveira, Alane Cabral Menezes de Oliveira, Ana Paula Esteves Pereira, Ana Paula Sayuri Sato, Antônio Augusto Moura da Silva, Carolina Abreu de Carvalho, Caroline de Barros Gomes, Claudia Leite de Moraes, Claudia Saunders, Cristina Garcia de Lima Parada, Daniela Saes Sartorelli, Dayana Rodrigues Farias, Denise Cavalcante de Barros, Denise Petrucci Gigante, Djanilson Barbosa dos Santos, Edson Theodoro dos Santos Neto, Elisa Maria de Aquino Lacerda, Elizabeth Fujimori, Fernanda Garanhani de Castro Surita, Flávia Farias Lima, Gilberto Kac, Helena Mendes Constante, Iracema de Mattos Paranhos Calderon, Isabel Oliveira Bierhals, Isaac Suzart Gomes-Filho, Jane de Carlos Santana Capelli, Jerusa da Mota Santana, José Guilherme Cecatti, Juliana dos Santos Vaz, Juraci Almeida Cesar, Katrini Guidolini Martinelli, Lívia Castro Crivellenti, Luana Patrícia Marmitt, Marco Fabio Mastroeni, Maria Angélica A Nunes, Maria Antonieta de Barros Leite Carvalhaes, Maria do Carmo Leal, Maria Inês Schmidt, Maria Fernanda Larcher de Almeida, Mayra Pacheco Fernandes, Michael Eduardo Reichenheim, Michele Drehmer, Mônica Araujo Batalha, Nathalia Cristina de Freitas-Costa, Patricia de Carvalho Padilha, Renato Passini Junior, Renato Teixeira Souza, Silmara Salete de Barros Silva Mastroeni, Silvia Regina Dias Medici Saldiva, Sílvio O M Prietsch, Simone Seixas da Cruz, Sirlei Siani Morais, Sotero Serrate Mengue, Thaís Rangel Bousquet Carrilho, Vera Lúcia Bosa

**Affiliations:** 6 Postgraduate Program in Collective Health, Federal University of Espírito Santo; 7 Postgraduate Program in Nutrition, Faculty of Nutrition, Federal University of Alagoas and Postgraduate Program of Health Science, Institute of Biological Sciences and Health, Federal University of Alagoas; 8 National School of Public Health, Oswaldo Cruz Foundation; 9 Department of Epidemiology, School of Public Health, University of São Paulo; 10 Postgraduate Program in Public Health, Federal University of Maranhão; 11 Postgraduate Program in Public Health, Botucatu Medical School, São Paulo State University; 12 Department of Epidemiology, Institute of Social Medicine Hesio Cordeiro, Rio de Janeiro State University; 40 Graduate Program in Family Health , Estácio de Sá University; 13 Department of Nutrition and Dietetics, Josue de Castro Nutrition Institute, Federal University of Rio de Janeiro; 14 Botucatu Medical School, São Paulo State University; 15 Postgraduate Program in Public Health; 41 Postgraduate Program in Nutrition and Metabolism, Department of Social Medicine, Ribeirao Preto Medical School, University of São Paulo; 16 Nutritional Epidemiology Observatory, Department of Social and Applied Nutrition, Josue de Castro Nutrition Institute, Federal University of Rio de Janeiro; 17 Department of Nutrition, Faculty of Nutrition, Federal University of Pelotas; 18 Health Sciences Center, Federal University of Recôncavo da Bahia; 19 Department of Nutrition and Dietetics, Josué de Castro Nutrition Institute, Federal University of Rio de Janeiro; 20 Public Health Nursing Department, School of Nursing, University of São Paulo; 21 Department of Obstetrics and Gynecology, School of Medical Sciences, University of Campinas; 22 Federal University of Rio de Janeiro, campus Macaé; 23 Department of Gynecology and Obstetrics, Botucatu School of Medicine, Paulista State University Júlio de Mesquita Filho; 24 Public Health Graduate Program, University of Southern Santa Catarina. Criciúma, Santa Catarina, Brazil; 25 Department of Health, Feira de Santana State University; 26 Postgraduate Program in Nutrition and Foods, Faculty of Nutrition, Federal University of Pelotas; 27 Postgraduate Program in Public Health, Federal University of Rio Grande; 28 Postgraduate Program in Public Health, Department of Social Medicine, Ribeirao Preto Medical School, University of São Paulo; 29 Postgraduate Program in Bioscience and Health, University of Western Santa Catarina; 30 Postgraduate Program in Health and Environment, University of Joinville Region; 31 Postgraduate Studies Program in Epidemiology, School of Medicine, Federal University of Rio Grande do Sul; 32 Department of Epidemiology and Quantitative Methods in Health, National School of Public Health, Oswaldo Cruz Foundation; 33 Postgraduate program in Epidemiology, Department of Social Medicine, Federal University of Pelotas; 34 Department of Epidemiology, Institute of Social Medicine, Rio de Janeiro State University; 35 Department of Obstetrics and Gynecology, School of Medicine, University of Campinas; 36 Health Sciences Center, University of the Joinville Region; 37 Hospital of Clinics, Faculty of Medicine, University of São Paulo; 38 Department of Epidemiology, Federal University of Recôncavo da Bahia; 39 Postgraduate Program in Food, Nutrition and Health, Department of Nutrition, Federal University of Rio Grande do Sul; 1 Nutritional Epidemiology Observatory, Josué de Castro Nutrition Institute, Federal University of Rio de Janeiro, Rio de Janeiro, Brazil; 2 Department of Obstetrics and Gynaecology, University of British Columbia, Faculty of Medicine, Vancouver, Canada; 3 Department of Sociological Studies, University of Sheffield, Sheffield, United Kingdom; 4 Department of Public Health Nursing, School of Nursing, University of São Paulo, São Paulo, Brazil; 5 Department of Epidemiology, University of São Paulo, São Paulo, Brazil

**Keywords:** hemoglobin, anemia, pregnancy, prevalence, Brazil, low-middle income countries

## Abstract

**Background:**

Anemia is common among pregnant women from low- and middle-income countries, but national estimates are scarce.

**Objectives:**

We aimed to assess hemoglobin (Hb) concentrations and the anemia prevalence in Brazil.

**Methods:**

Data included 12,287 pregnant women aged 15–49 y and 17,967 measurements from 7 studies (2007–2014) participating in the Brazilian Maternal and Child Nutrition Consortium. Hb (g/dL) was obtained from medical records (6 studies, 17,565 measurements) or capillary blood samples (1 study, 402 measurements). Hb <11, <10.5, and <11 g/dL were used to define anemia at the first, second, and third trimesters using the 2024 WHO guideline. Identification of implausible Hb values and heterogeneity analysis were performed. We estimated medians and interquartile ranges for the Hb concentration and prevalence and 95% confidence intervals (CI) of anemia according to maternal prepregnancy BMI, age, and education, gestational trimester, and year of data collection.

**Results:**

Median Hb was 12.0 (95% CI: 11.2, 12.8) g/dL; no differences were observed according to the studied covariables. Anemia prevalence was 14.1% (95% CI: 13.6, 14.6), and highest during 2013 [27.9% (95% CI: 17.3%, 38.6%)] and in the third trimester [23.5% (95% CI: 22.5%, 24.6%)]. Higher anemia prevalence in the third compared with the first trimester was also observed among women aged 15–19 [1^st^: 8.02% (95% CI: 6.2%, 9.9%); 3^rd^: 28.1% (95% CI: 25.4%, 30.8%)] than those aged 20–49 y [1^st^: 6.5%; (5.8%, 7.2%); 3^rd^: 22.6% (95% CI: 21.4%, 23.7%)]. Anemia prevalence for those with education ≤4 y (15.9%; 95% CI: 14.1%, 17.8%) and women with prepregnancy underweight (19.2%; 95% CI: 15.9%, 22.4%) and normal weight (15.3%; 95% CI: 14.4%, 16.2%) were higher than those with 9–11 (13.1%; 95% CI: 12.4%, 13.8%) and 12–18 y (10.3%; 95% CI: 9.2%, 11.0%), and overweight (12.2%; 95% CI: 10.8%, 13.6%) and obesity (9.9%; 95% CI: 8.1%, 11.7%).

**Conclusions:**

Anemia was higher in adolescents compared with older women and in the third trimester compared with the first, underscoring the need for targeted monitoring during these periods.

## Introduction

During pregnancy, normal physiological changes cause relative or absolute reductions in hemoglobin (Hb) concentration, which makes anemia one of the most frequent complications in this period [1,2]. According to the FAO of the United Nations, in low and middle-income countries, risk of anemia is higher among children and women of reproductive age, especially among those suffering from food insecurity [3]. Poverty and inequities, including the lack of access to health services and food and nutrition security, appear to be important determinants of the increased risk in these countries [4]. Anemia during pregnancy is associated with maternal and fetal morbidity and mortality. Studies show that anemia can increase risk of hemorrhage and maternal morbidity, and death during pregnancy, preterm birth, low birth weight, and infant death [1,2].

In Brazil, the Ministry of Health recommends maternal micronutrient supplementation during pregnancy to reduce risk of anemia [5]. Since 2005, the recommendation has been to supplement iron and folic acid during pregnancy, independent of a biochemical evaluation of the individual’s status. The supplementation program was updated in 2022 [5], and the current recommendation is that pregnant women should be supplemented daily with 40 mg elemental iron after pregnancy confirmation until the end of pregnancy and 0.4 mg folic acid up to the 12^th^ wk of pregnancy for the prevention of neural tube defects [6].

Despite the recent changes in the national supplementation guidelines, there are no national Brazilian data on the prevalence of maternal anemia or the distribution of Hb concentrations during pregnancy. Preliminary data from studies participating in the Brazilian Maternal and Child Nutrition Consortium (BMCNC) were used to support this review. In addition, in 2024, the WHO proposed new cutoff points for defining anemia by gestational trimester [7]. Estimating the prevalence of maternal anemia during pregnancy is of pivotal importance to establishing public health policies aiming to reduce this condition, potentially impacting maternal morbidity and mortality. Thus, we aimed to characterize the distribution of Hb concentrations and the prevalence of anemia among Brazilian pregnant women from the studies participating in the BMCNC.

## Methods

### Data source and sample

We used data from the BMCNC, a network created in 2019 to harmonize multiple datasets across Brazil containing data on pregnant women and their children. The first phase of the BMCNC focused on developing new Brazilian gestational weight gain charts [8]. In the second phase, efforts were made to obtain new datasets and analyze other essential pregnancy markers such as symphysis fundal height, blood pressure, anemia, and dietary intake. Details about the establishment, data harmonization, and basic characteristics of the BMCNC studies can be found elsewhere [9].

The BMCNC currently includes 38 datasets from studies conducted in Brazil between 1990 and 2018. We identified 12 datasets containing maternal Hb concentrations during pregnancy among those studies. These datasets were individually cleaned and harmonized. After this stage, 4 datasets were removed from the pool because they did not include the gestational age of the Hb assessment. Then, implausible values were identified and removed, and the heterogeneity of the Hb data across the studies determined the elimination of an additional dataset (methods described in detail below). Individuals between 15 and 49 y with Hb measurements collected during pregnancy and their respective gestational ages were eligible.

### Hb and anemia

Hb (g/dL) was obtained from medical records (*n =* 7) or capillary blood samples using the portable Hb analyzer—Hemocue (*n =* 1). Hb data obtained from medical records were assumed to be derived from Hb venipuncture measurements [10]. Hb values <2 and >16 were excluded because they were considered biologically implausible. The presence of anemia was defined if the Hb values at the first, second, and third trimesters were < 11, <10.5, and <11 g/dL, respectively [7]. In most studies, multiple Hb records for the same individual throughout pregnancy contributed data across >1 gestational trimester.

### Other variables

Gestational age in each prenatal care visit (in which Hb was collected) was calculated based on an ultrasound performed before 24 wk of gestation. If ultrasound data were unavailable or the examination was conducted after 24 wk, gestational age was calculated based on the last menstrual period date. Gestational age at the time of Hb concentration assessment was classified into trimesters as follows: 1st trimester—≤13 wk and 3 d (∼94 d); 2nd trimester—from 13 wk and 4 d (∼95 d) to 26 wk and 3 d (∼185 d); and 3rd trimester—from 26 wk and 4 d (∼186 d) until delivery [10]. We compared the Hb concentration according to last menstrual period and ultrasound for gestational trimesters and throughout gestation and found no differences (Supplemental Figures 1 and 2).

Maternal age was assessed in years and categorized into 15–19, 20–24, 25–34, and 35–49. Maternal education was recorded in years of completed study and classified as 0–4, 5–8, 9–11, and 12–18. Prepregnancy body mass index (BMI) was calculated based on self-reported or measured first trimester (≤8 wk) weight (kg) divided by height (m^2^). For adults (age > 19 y), BMI (kg/m^2^) was classified using the WHO cutoffs as underweight (< 18.5), normal weight (≥ 18.5 and < 25.0), overweight (≥ 25.0 and < 30.0), and obesity (≥ 30) [11]. For adolescents (15–19 y o), BMI-for-age *z*-scores were calculated according to the WHO standards [12] and classified according to the cutoffs proposed for the Brazilian Food and Nutrition Surveillance System [13].

Other variables were extracted from the original databases and included the study collection’s macroregion (North, Northeast, Southeast, South, and Mid-West), year of data collection, and gestational trimester (first, second, and third).

### Statistical analysis

#### Identification of implausible values

We applied 2 cross-sectional methods to identify implausible Hb values in the sample. First, we calculated *z*-scores of Hb according to gestational age using the International Fetal and Newborn Growth Consortium for the 21st Century (INTERGROWTH-21st) Hb standards [14]. Second, we calculated the *z*-scores of the sample distribution of Hb by trimester. For both methods, values < −4 and > +4 *z*-scores were flagged as implausible and removed from the sample (Supplemental Figure 3).

### Heterogeneity analysis

Heterogeneity analysis was performed to verify whether the Hb distribution was homogeneous across the 8 datasets. First, as an exploratory step, we adjusted a multilevel model for Hb according to gestational age and the study of origin. From this model, we calculated the percentage of Hb variance that could be explained by the study of origin [15].

We calculated the standardized site difference (SSD) by subtracting the mean of Hb from each dataset in gestational age intervals (0–13, 14–18, 19–23, 24–28, 29–33, and 34–43 gestational wk) from the pooled mean for each interval and dividing by the pooled SD [9,16]. The datasets were considered homogenous if the SSDs for all intervals were −0.5/+0.5 [17]. For this analysis, datasets with *n* < 30 in each gestational age interval were not considered in the SSD calculation. In this stage, 1 dataset was excluded. Thus, this study included data from 7 original datasets that were harmonized and found to be homogeneous.

#### Descriptive analysis

The distribution of variables was assessed using histograms and scatter plots. We estimated medians and IQR for Hb concentration during pregnancy due to the lack of a normal distribution in this variable. Prevalence and 95% confidence intervals (CI) for anemia were estimated. These analyses were performed for the whole sample and according to maternal prepregnancy BMI, age, education, gestational trimester, and year of data collection.

We also constructed graphs portraying the Hb concentration throughout pregnancy compared with the INTERGROWTH-21st Hb standards [14]. The values for the centiles per week in these curves were calculated based on the equations available by Ohuma et al. [14]. Finally, we performed a sensitivity analysis for all the stratifications, excluding the data from the study using Hb collected from capillary blood samples. The analyses were performed in Stata (version 15) and R (version 4.0.2) statistical software in a Jupiter hub environment.

### Ethics

This study was approved by the Research Ethics Committee of the Rio de Janeiro Federal University Maternity Teaching Hospital (protocol number: 33897420.4.0000.5275). All the analyses were conducted using deidentified data. Each study from the BMCNC was approved by their institutional research ethics committees and conducted according to the principles of the Declaration of Helsinki. The de-identified datasets from each study were uploaded to a secure repository and accessed and analyzed by authorized researchers.

## Results

**FIGURE 1:**
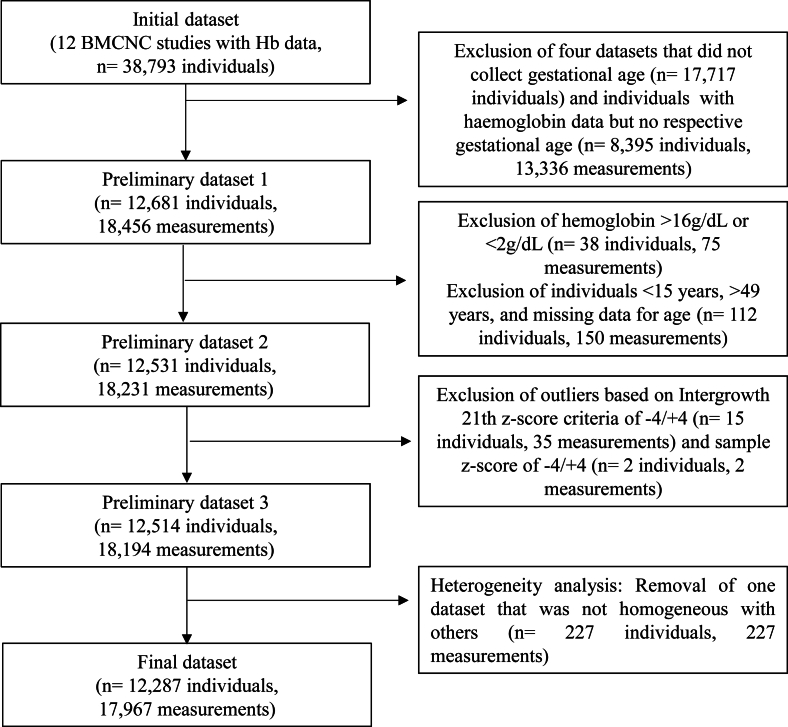
Flowchart for the constitution of the dataset used to describe the concentrations of hemoglobin and the prevalence of anemia during pregnancy, data from the Brazilian Maternal and Child Nutrition Consortium (BMCNC). Notes: Intergrowth-21st: International Fetal and Newborn Growth Consortium for the 21st Century.

The 8 eligible datasets from the BMCNC included 12,681 individuals and 18,456 Hb measurements with gestational age data. In the outlier assessment step, 17 individuals and 37 measurements were flagged and removed. Finally, after heterogeneity assessment, the 7 datasets included in this analysis comprised 12,287 individuals and 17,967 measurements (Figure 1).

Hb data were highly homogeneous according to heterogeneity assessment. The distribution of Hb among datasets was assessed according to gestational age intervals. All SSD values were within ±0.5 SD (Supplemental Figure 4). Between 2007 and 2014, most studies were conducted in the Southeast region, 1 in the 5 Brazilian macroregions, and 1 only in the Northeast region. Six used data from medical records, and 1 used capillary blood to assess Hb data (Table 1) [[18], [19], [20], [21], [22], [23], [24]].

**TABLE 1:** Description of studies included in the Brazilian Maternal and Child Nutrition Consortium with hemoglobin data for pregnant women with respective gestational age

First author (y)	Study name	Macroregion	Year of the data collection	Age (y)	Gestational age (wk)		Sample size with Hb data	Longitudinal data	*n* (%) with longitudinal	Range of Hb measurements across pregnancy	Total number of Hb measurements during pregnancy	Hb data source	Study population
				Median (min–max)	Median (IQR)	(min–max)							
Marano, 2012 [18]	PQ	SE	2007–2008	24.0 (15–44)	22.0 (14.6–31)	(4–43)	1037	Yes	464 (44.7)	1–2	1501	Medical records	Low-risk/basic healthcare
Santos-Neto, 2012 [19]	RMGV	SE	2010	23.5 (15–45)	19.6 (12.3–29.4)	(4–41)	489	Yes	232 (47.4)	1–3	730	Medical records	Low-risk/basic healthcare
Leal, 2012 [20]	BB	All	2011–2012	25.0 (15–49)	19.1 (11.1–29.9)	(4–43)	9,411	Yes	4358 (46.3)	1–2	13,769	Medical records	Any level of complexity and risk
Farias, 2013 [21]	RJ	SE	2011–2012	26.0 (19–40)	20.0 (11.6–26.8)	(4–42)	236	Yes	170 (72.0)	1–3	507	Medical records	Low-risk/basic healthcare
Polgliani, 2014 [22]	ES2	SE	2010–2011	24.0 (15–41)	21.4 (12.4–30.7)	(4–41)	216	Yes	122 (56.5)	1–3	352	Medical records	Any level of complexity and risk
Martinelli, 2014 [23]	ES1	SE	2012–2013	24.0 (15–42)	17.4 (11.6–29.0)	(4–42)	496	Yes	208 (41.9)	1–3	706	Medical records	Any level of complexity and risk
Ferreira, 2020 [24]	AL	NE	2014	23.0 (15–43)	24.0 (16.0–31.4)	(5–42)	402	No	0 (0.0)	1–1	402	Capillary blood sample	Low-risk / basic healthcare

Abbreviations: Hb, hemoglobin; NE, northeast; SE, southeast.

Studies names are derivated from acronyms and abbreviations from Portuguese: *PQ*, Petrópolis e Queimados; *RMGV*, Região Metropolitana da Grande Vitória; *BB*, Nascer no Brasil; *RJ*, Rio de Janeiro; *ES1*, Espírito Santo 1; *ES2*, Espírito Santo 2; *AL*, Alagoas.

**FIGURE 2:**
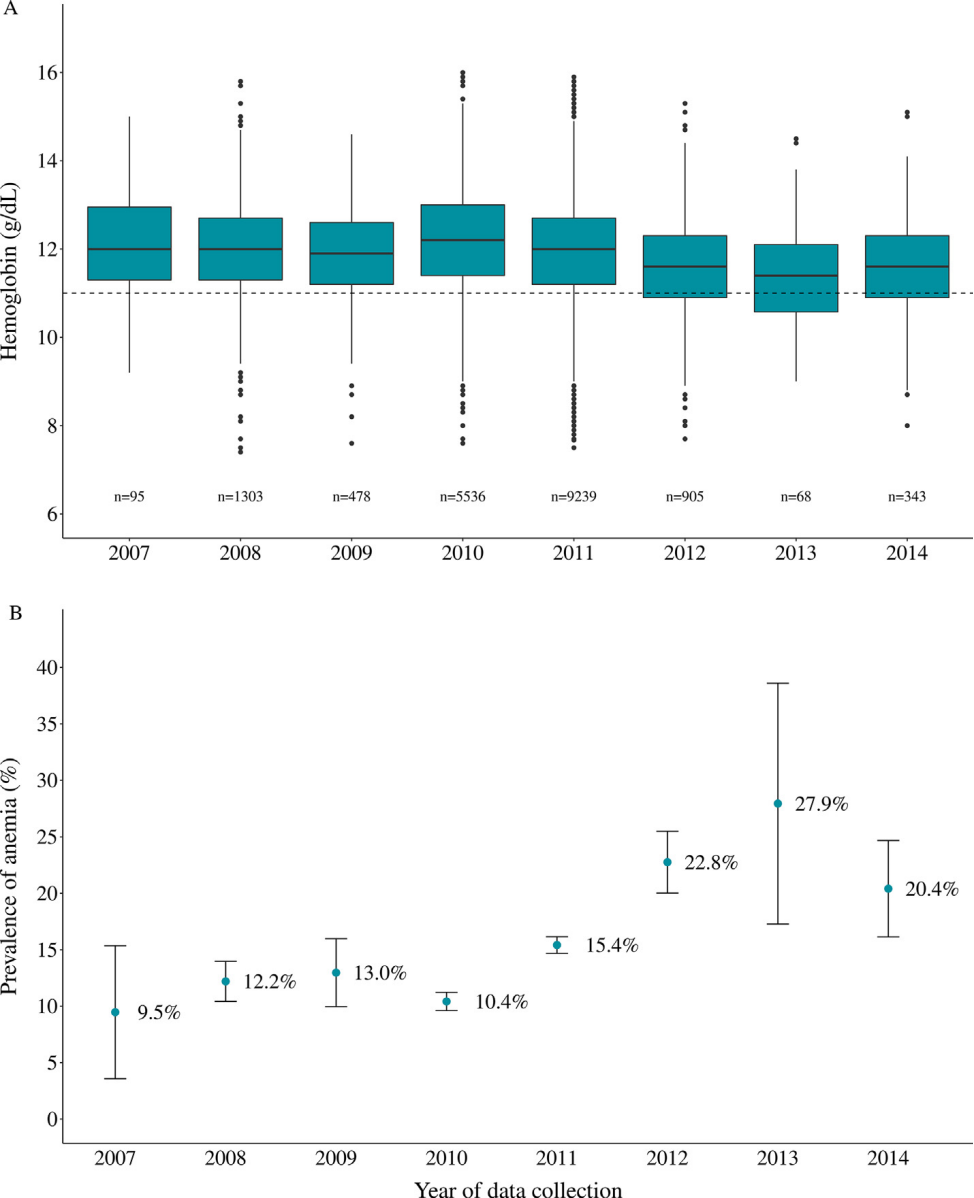
Distribution of maternal hemoglobin (A) in g/dL and prevalence and 95% confidence intervals for anemia (B) during pregnancy according to year of the data collection. Note: dashed line indicates Hb=11 g/dL. Anemia was defined according to WHO threshold: Hb < 11, <10.5, and <11 g/dL at 1st, 2nd, and 3rd trimesters, respectively [7].

The median Hb concentration was 12.0 g/dL (IQR: 11.2–12.8). No differences were observed between the median Hb according to the selected covariables (Figure 2A and Table 2). Nonetheless, the highest prevalence of anemia was observed in 2012–2014 (2012: 22.8%, 95% CI: 20.0%, 25.5%; 2013: 27.3%, 95% CI: 17.3%, 38.6%; 2014: 20.4%, 95% CI: 16.1%, 24.7%), and the lowest in 2007 (9.5%, 95% CI: 3.6%, 15.4%) (Figure 2).

**TABLE 2:** Distribution of the concentrations of hemoglobin (g/dL) and the prevalence of anemia^6^ according to maternal characteristics (*n =* 17,967)

Variables	Distribution of hemoglobin		Prevalence of anemia	
	Median (IQR)	Range (min–max)	% (*n*)	95% CI
Overall	12.0 (11.2–12.8)	7.4–16.0	14.1 (2527)	13.6, 14.6
Maternal age (y) (*n =* 12,287)				
15–19	11.8 (11.0–12.6)	7.40–16.0	17.4 (562)^1^^,^^2^^,^^3^	16.1, 18.7
20–24	12.0 (11.2–12.7)	7.50–15.8	14.9 (764)^1^^,^^4^^,^^5^	13.9, 15.8
25–34	12.1 (11.3–12.9)	7.70–16.0	12.4 (968)^2^^,^^4^	11.7, 13.1
35–49	12.1 (11.1–12.9)	7.50–15.4	12.9 (233)^3^^,^^5^	11.4, 14.5
Maternal education (schooling years) (*n =* 12,242)				
0–4	11.9 (11.1–12.7)	7.70–15.7	15.9 (244)^1^^,^^2^	14.1, 17.8
5–8	11.9 (11.1–12.6)	7.40–16.0	16.8 (907)^3^^,^^4^	15.8, 17.8
9–11	12.0 (11.3–12.8)	7.70–16.0	13.1 (1083)^1^^,^^3^^,^^5^	12.4, 13.8
12–18	12.2 (11.5–12.9)	7.70–16.0	10.3 (281)^2^^,^^4^^,^^5^	9.2, 11.5
Prepregnancy BMI^7^ (*n =* 6596)				
Underweight	11.8 (11.0–12.6)	8.70–14.8	19.2 (106)^1^^,^^2^	15.9, 22.4
Normal	12.0 (11.2–12.7)	7.50–16.0	15.3 (908)^3^^,^^4^	14.4, 16.2
Overweight	12.1 (11.3–12.9)	7.90–15.4	12.2 (261)^1^^,^^3^	10.8, 13.6
Obesity	12.2 (11.4–13.0)	7.50–15.8	9.9 (103)^2^^,^^4^	8.1, 11.7
Gestational trimester (*n =* 12,287)				
First (4–13 wk)	12.6 (11.9–13.2)	8.40–16.0	6.7 (390)^1^^,^^2^	6.1, 7.4
Second (14–29 wk)	11.8 (11.1–12.5)	7.50–16.0	11.4 (673)^1^^,^^3^	10.5, 12.2
Third (27–43 wk)	11.7 (11.0–12.4)	7.40–15.9	23.5 (1464)^2^^,^^3^	22.5, 24.6
Macroregion (*n =* 12,287)				
North	12.0 (11.1–12.7)	7.67–16.0	15.2 (185)^1^	13.1, 17.2
Northeast	12.0 (11.1–12.7)	7.90–16.0	15.6 (562)^2^	14.4, 16.8
Southeast	12.0 (11.2–12.8)	7.40–16.0	14.1 (1249)^3^	13.3, 14.8
South	12.0 (11.2–12.8)	7.80–16.0	13.8 (450)^4^	12.6, 15.0
Mid-West	12.3 (11.6–13.2)	8.10–15.7	8.2 (81)^1^^,^^2^^,^^3^^,^^4^	6.5, 9.9

Abbreviation: CI, confidence interval.

1,2,3,4,5 superscript numbers represent differences between the variables categories using lack of overlapping 95% CI.

6 Anemia was defined according to WHO threshold, Hb < 11, <10.5, and <11 g/dL at 1st, 2nd, and 3rd trimesters, respectively [7].

7 BMI was classified differently for adolescents and adults, according to WHO. For adults (age > 19 y), BMI (kg/m^2^) was classified as underweight (<18.5), normal weight (≥18.5 and <25.0), overweight (≥25.0 and <30.0), and obesity (≥30) [11]. For adolescents (age ≤19 y), BMI-for-age *z*-scores were calculated considering the women’s age according to WHO thresholds [12] and classified according to the cutoffs proposed for the Brazilian Food and Nutrition Surveillance System [13].

The prevalence of anemia was 14.1% (95% CI: 13.6%, 14.6%). The highest prevalence was observed for women aged 15–19 (17.4%, CI: 16.1%, 18.7%) and 20–24 (14.9%, CI: 13.9%, 15.8%), and among those with education ≤ 4 y (15.9%; 95% CI: 14.1%, 17.8%). We also observed higher anemia prevalence among women with prepregnancy underweight (19.2%; 95% CI: 15.9%, 22.4%) and normal weight (15.3%; 95% CI: 14.4%, 16.2%) compared with those with overweight (12.2%; 95% CI: 10.8%, 13.6%) and obesity (9.9%; 95% CI: 8.1%, 11.7%) (Table 2).

**FIGURE 3:**
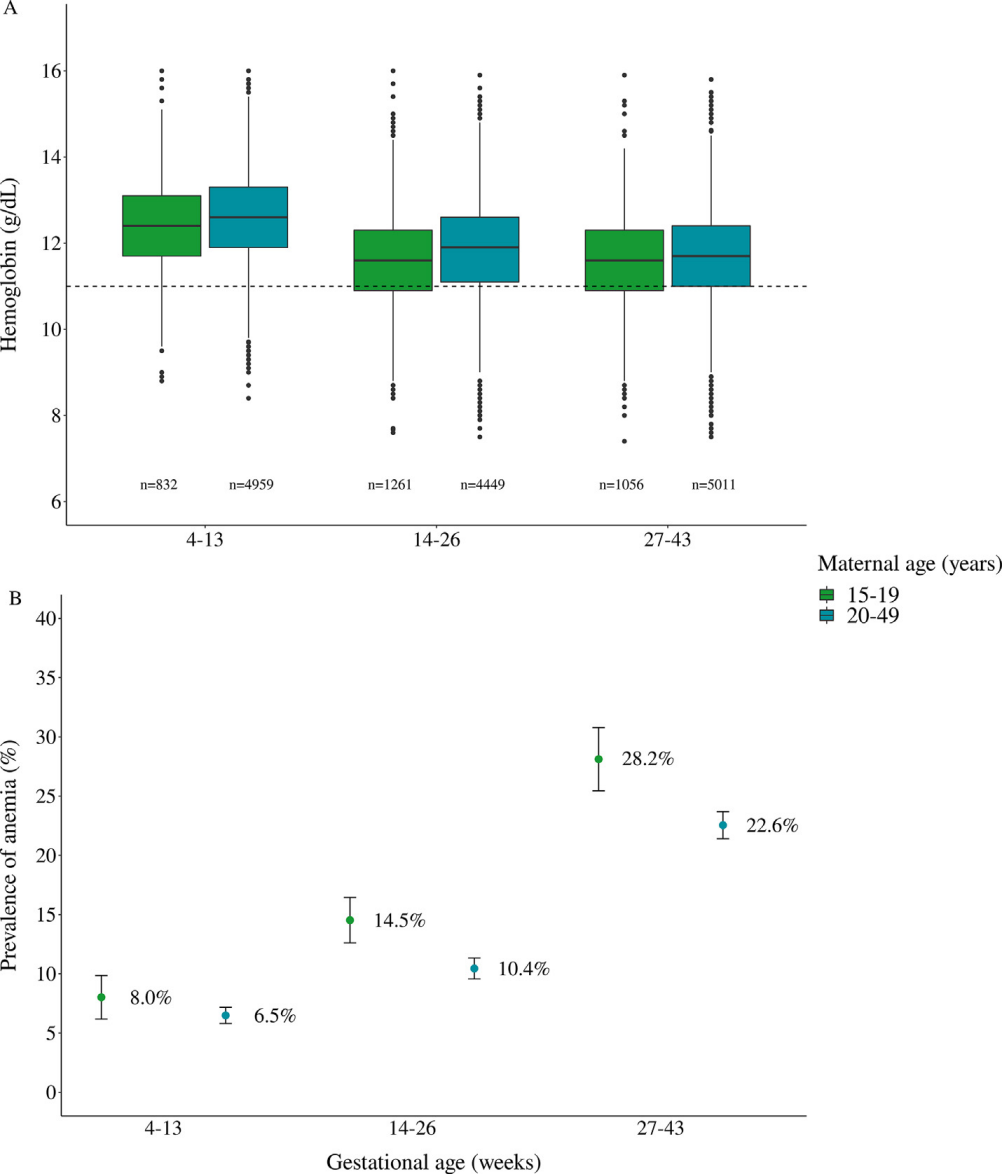
Distribution of maternal hemoglobin (A) in g/dL and prevalence and 95% confidence intervals for anemia (B) during pregnancy according to gestational age and maternal age. Note: dashed line indicates Hb=11. Anemia was defined according to WHO threshold: Hb < 11, <10.5, and <11 g/dL at 1st, 2nd, and 3rd trimesters, respectively [7].

The prevalence of anemia was 6.7% (95% CI: 6.1%, 7.4%), 11.3% (95% CI: 10.5%, 12.1%), and 23.5% (95% CI: 22.5%, 24.6%) at the first, second, and third gestational trimester, respectively (Table 2). The highest prevalence of anemia observed between the first and third trimesters was noticed when analysis was stratified by age group, that is, higher value was observed in third trimester among women 15–19 age group (1st: 8.02; 95% CI: 6.2, 9.9; 3rd: 28.1; 95% CI: 25.4, 30.8) compared with 20–49 y (1st: 6.5; 95% CI: 5.8, 7.2; 3rd: 22.6; 95% CI: 21.4, 23.7). Although the median of Hb did not show a significant difference between age groups, the Hb concentration exhibited the same downward trend (Figure 3).

**FIGURE 4:**
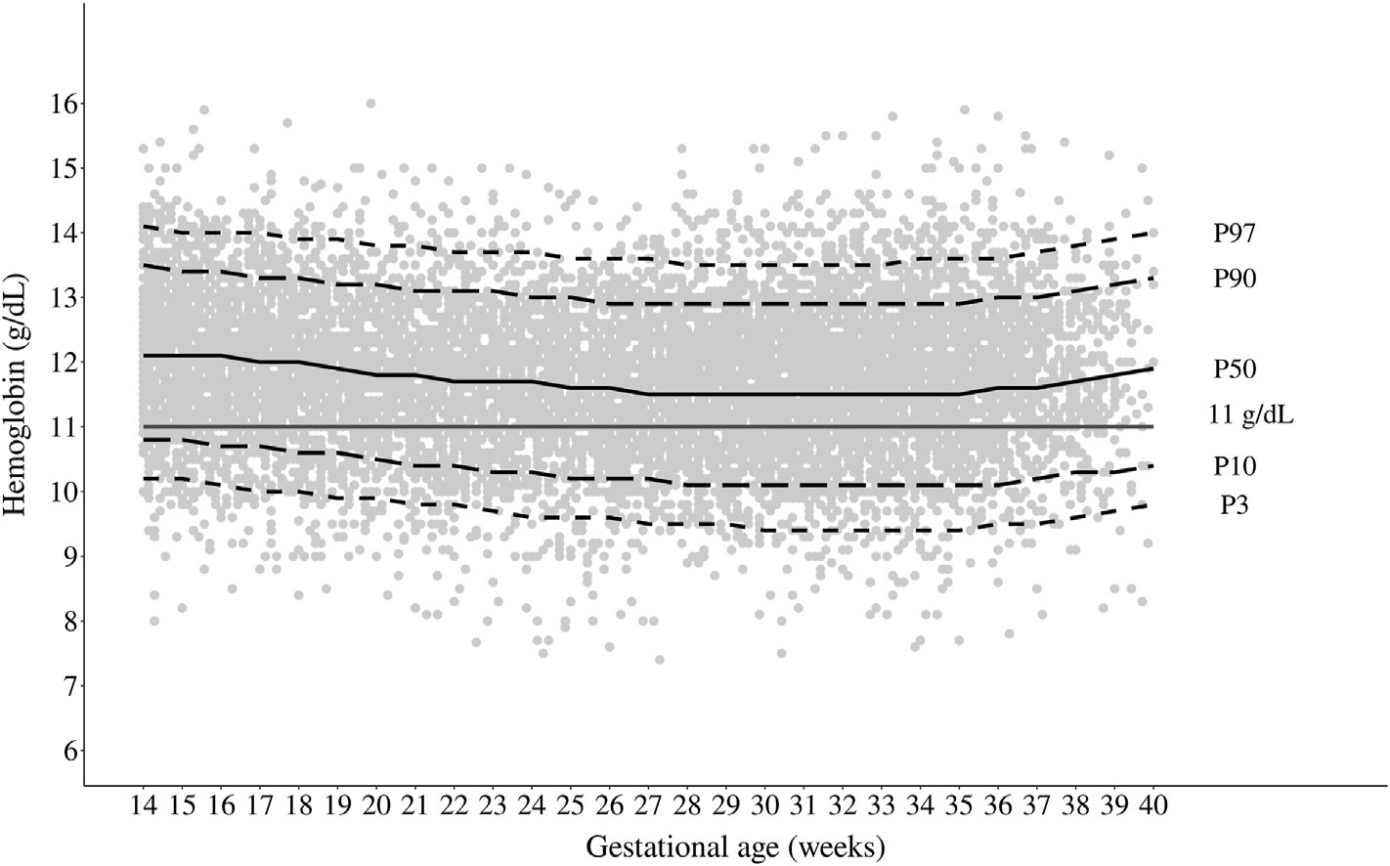
Distribution of hemoglobin (g/dL) during pregnancy according to the percentiles of data distribution and the International Fetal and Newborn Growth Consortium for the 21st Century (INTERGROWTH-21st) charts [14]. Note: line at 11 g/dL refers to the cutoff for diagnosing anemia in 1^st^ and 3^rd^ gestational trimester according to the WHO. In the 2^nd^ gestational trimester, the cutoff for diagnosing anemia is 10.5 g/dL [7].

Hb measurements distribution throughout pregnancy were not concentrated in specific gestational ages, and most measurements were within the 3rd and 97th percentiles of the INTERGROWTH-21st charts. Throughout pregnancy, 46.3% of the measurements were below the 50th percentile of the charts, and 9.8% were <10th and 88.1% <90th percentiles (Figure 4).

When we performed the sensitivity analysis excluding the study using Hb obtained from capillary blood samples, no differences were observed in the concentrations of Hb or prevalence of anemia in general (data not shown in tables) or stratified by the selected variables (Supplemental Table 1 and Supplemental Figures 5, 6, and 7).

## Discussion

We observed a high prevalence of anemia among Brazilian women. According to WHO standards, the prevalence of anemia is considered mild overall but is classified as moderate during adolescence and the third trimester in this Brazilian group of women of reproductive age. Women with underweight and normal weight prepregnancy and low education also had a higher prevalence of anemia. The BMCNC findings also show that the highest prevalence of anemia was observed in the third pregnancy trimester, and between 2012 and 2014, the last years of our time series.

The current results represent the first publication of national data on the prevalence of anemia among pregnant women according to the Brazilian macroregions and gestational trimesters. Biete et al. [25] metaanalyzed data from 73 Brazilian studies and 12,792 individuals conducted between 1988 and 2020 and estimated a prevalence of anemia of 23% at any time during pregnancy [25]. In this study, we estimated the prevalence of anemia by gestational trimester and observed higher values at the end of pregnancy. The value observed in the third trimester was virtually identical to that reported by Biete et al. [25] (23.5% in the BMCNC compared with 22.4%). In the Americas, the prevalence of anemia at any time during pregnancy is estimated to be 22.4% [4]. It is essential to highlight the need for country-specific estimates. The American continent exhibits significant differences in income levels and epidemiological and nutritional transition stages, affecting access to health services and adequate nutrition. This could translate into a very different prevalence of anemia across countries [26,27].

As expected, we observed an increase in the prevalence of anemia from the first to the third trimester, which was almost threefold [28,29]. Worldwide studies also report a higher prevalence of anemia in the third trimester [30,31]. This may be related to the rapid growth of the fetus in the second and third trimesters and the significant increase in demand for nutrients such as iron [32,33]. Recently, the WHO proposed new Hb cutoffs to define anemia, changing it in the second trimester to 10.5 g/dL but keeping <11 g/dL for the other trimesters. Previously, the anemia threshold was Hb <11 g/dL at any time during pregnancy. The second trimester is critical, as Hb levels in the blood will be lower due to hemodilution [29]. We considered the new cutoff points for this manuscript and observed an increase in anemia prevalence between the first and second and between the second and third trimesters.

We also observed an increase in the prevalence of anemia between 2007 and 2012. According to our findings, after 2012, the estimates appear to have stabilized. Araujo Costa et al. [4] used the WHO dataset and revealed that most countries in the Americas had a reduction of <1% or an increase in the estimates of the prevalence of anemia among pregnant women aged 15–49 y [4] between 2015 and 2019, a result that aligns with the current study. However, Steves et al. [34] reported a reduction in the prevalence of anemia from 29% to 22% among pregnant women in Latin America [34]. This falls short of the WHO’s target of reducing this nutritional problem by 50% by 2025 and the sustainable development goals (SDG) that aim to ensure healthy lives and promote well-being for all, which include reducing anemia by 2030 [35]. In addition, these rates appear to have stabilized because the definition of SDG, indicating the need to evaluate public policies that seek to improve this situation [4,36].

In our study, women with lower education levels (0–4 and 5–8 schooling years) had higher prevalence of anemia, and we also observed lower levels of anemia in higher education levels. Socioeconomic status and poverty appear to be one of the main determinants of anemia [4,37]. Individuals with low socioeconomic status are at greater risk of exposure to anemia and its sequelae [38]. This is probably due to access to better living conditions in general, including the guarantee of adequate nutrition and health education [39]. Our findings are in line with other studies that have observed higher anemia prevalence among women with lower education levels in Brazil and other low- and middle-income countries [[40], [41], [42], [43]].

We also observed that 17.4% of pregnant adolescents (15–19 y) had anemia at some point during pregnancy, and an increase in prevalence from the first to the third trimester from 8.0% to 28.2%. During adolescence, nutritional needs increase significantly due to rapid somatic growth, increased erythrocyte mass, and the onset of menstruation [44]. In addition to the nutritional demands of adolescence, the nutritional requirements of pregnancy place adolescents in a high-risk group. National data for adolescents are scarcer than for adults. A population survey conducted in the Brazilian Amazon also demonstrated an increased anemia risk immediately after delivery among women <19 compared with those ≥ 19 y [40].

Prepregnancy or early pregnancy nutritional status is another determinant of anemia. In our study, anemia was higher among underweight and normal-weight individuals. Low early pregnancy weight or BMI may be a reflection of deficient nutritional intake, including intake of micronutrients that are essential for hematopoiesis [37]. In addition, being underweight may result from multiple infections or reinfections, such as parasites, which subsequently lead to anemia, a common problem in developing countries [1,45]. We also observed lower anemia prevalence among individuals with overweight or obesity. A study of 1379 women in Ghana and Indonesia demonstrated that lower BMI in early pregnancy was associated with higher Hb levels, and the anemia risk decreased with higher early pregnancy BMI [46]. Results of a meta-analysis that included data from 83,554 participants reinforce that underweight prepregnancy BMI leads to a risk of iron deficiency, whereas excess prepregnancy BMI does not [47].

This is the first study to present national data on anemia during pregnancy in Brazil, and the availability of data for each gestational trimester is worth mentioning. In addition, the careful data harmonization process, including steps of outliers and heterogeneity assessment planned a priori, makes the results of this study valuable for evaluating the public policies in place in the country. The Brazilian iron supplementation program is almost 20 y old; however, the review of this program would not have been possible without the national data on anemia prevalence in pregnant individuals. The Brazilian Ministry of Health used early reports based on data from BMCNC that were prepared exclusively to review this supplementation program. On the basis of this evaluation, the decision was to maintain iron supplementation throughout pregnancy, considering anemia is still a mild-to-moderate public health problem for this population.

However, our study has limitations. Variables such as parity/interpregnancy interval, use of supplements, or adherence to the national supplementation program were unavailable for most of the included studies. The use of Hb data from medical records for most studies is another significant limitation. Although it is safe to assume that Hb concentrations were obtained from venipuncture, the collection of the samples was not standardized across the studies. In this sense, the heterogeneity assessment results helped reinforce the homogeneity of the data and likely of the data collection method, indicating a similar measurement error. Finally, the selected studies included in this analysis are concentrated in the country’s Southeast region. However, previous studies have shown that the profile of the women participating in the BMCNC studies is similar to that of the Brazilian population in general [9].

In conclusion, based on data from 2000 to 2014, anemia during pregnancy was shown to still be a public health problem in Brazil. National estimates are essential when evaluating supplementation programs and shedding light on the most vulnerable groups. Individuals with lower prepregnancy BMI, adolescents, those with lower education, and those in the third trimester of pregnancy require greater attention during prenatal care to help minimize adverse outcomes related to anemia.

## Author contributions

NCFC, HMC, and TRBC analyzed and interpreted the data and wrote the manuscript, with input from all authors. PNC, EF, and APSS contributed to the study conception and design, the interpretation of the data, and the revision of the manuscript. BMCNC contributed to the interpretation of the data and revision of the manuscript. GK is the coordinator of the Brazilian Maternal and Child Nutrition Consortium and participated in all phases of analysis and interpretation of the data and writing of the manuscript. All authors read and approved the final version of the manuscript.

## Data Availability

The BMCNC is managed by the team of researchers from the Nutritional Epidemiology Observatory, from the Nutrition Institute, in the Federal University of Rio de Janeiro. Datasets are not yet available for public use, but requests can be made to the coordinator of the project (gilberto.kac@gmail.com) or through the project data repository (https://dataverse.nutricao.ufrj.br/dataverse/conmai_openaccess) and the whole consortium group is consulted regarding data sharing for specific studies.

## Funding

The Brazilian National Research Council (CNPq) provided financial support for this study on process n. 402576/2021-7, and Rio de Janeiro State Research Support Foundation (FAPERJ) on process n. 260003/015750-2021. TRBC is a Michael Smith Health Research BC Research Trainee (RT-2022-2582).

## Conflict of interest

GK reports financial support was provided by the Brazilian National Research Council (CPNq) and Rio de Janeiro State Research Support Foundation (FAPERJ). *All other authors declare* no potential conflicts of interest.

## Members of the Brazilian Maternal and Child Nutrition Consortium

Adauto Emmerich Oliveira (Postgraduate Program in Collective Health, Federal University of Espírito Santo), Alane Cabral Menezes de Oliveira (Postgraduate Program in Nutrition, Faculty of Nutrition, Federal University of Alagoas and Postgraduate Program of Health Science, Institute of Biological Sciences and Health, Federal University of Alagoas), Ana Paula Esteves Pereira (National School of Public Health, Oswaldo Cruz Foundation), Ana Paula Sayuri Sato (Department of Epidemiology, School of Public Health, University of São Paulo), Antônio Augusto Moura da Silva (Postgraduate Program in Public Health, Federal University of Maranhão), Carolina Abreu de Carvalho (Postgraduate Program in Public Health, Federal University of Maranhão), Caroline de Barros Gomes (Postgraduate Program in Public Health, Botucatu Medical School, São Paulo State University), Claudia Leite de Moraes (Department of Epidemiology, Institute of Social Medicine Hesio Cordeiro, Rio de Janeiro State University; Graduate Program in Family Health , Estácio de Sá University), Claudia Saunders (Department of Nutrition and Dietetics, Josue de Castro Nutrition Institute, Federal University of Rio de Janeiro), Cristina Garcia de Lima Parada (Botucatu Medical School, São Paulo State University), Daniela Saes Sartorelli (Postgraduate Program in Public Health; Postgraduate Program in Nutrition and Metabolism, Department of Social Medicine, Ribeirao Preto Medical School, University of São Paulo), Dayana Rodrigues Farias (Nutritional Epidemiology Observatory, Department of Social and Applied Nutrition, Josue de Castro Nutrition Institute, Federal University of Rio de Janeiro), Denise Cavalcante de Barros (National School of Public Health, Oswaldo Cruz Foundation), Denise Petrucci Gigante (Department of Nutrition, Faculty of Nutrition, Federal University of Pelotas), Djanilson Barbosa dos Santos (Health Sciences Center, Federal University of Recôncavo da Bahia), Edson Theodoro dos Santos Neto (Postgraduate Program in Collective Health, Federal University of Espírito Santo), Elisa Maria de Aquino Lacerda (Department of Nutrition and Dietetics, Josué de Castro Nutrition Institute, Federal University of Rio de Janeiro), Elizabeth Fujimori (Public Health Nursing Department, School of Nursing, University of São Paulo), Fernanda Garanhani de Castro Surita (Department of Obstetrics and Gynecology, School of Medical Sciences, University of Campinas), Flávia Farias Lima (Federal University of Rio de Janeiro, campus Macaé), Gilberto Kac (Nutritional Epidemiology Observatory, Department of Social and Applied Nutrition, Josue de Castro Nutrition Institute, Federal University of Rio de Janeiro), Helena Mendes Constante (Nutritional Epidemiology Observatory, Department of Social and Applied Nutrition, Josue de Castro Nutrition Institute, Federal University of Rio de Janeiro), Iracema de Mattos Paranhos Calderon (Department of Gynecology and Obstetrics, Botucatu School of Medicine, Paulista State University Júlio de Mesquita Filho), Isabel Oliveira Bierhals (Public Health Graduate Program, University of Southern Santa Catarina. Criciúma, Santa Catarina, Brazil), Isaac Suzart Gomes-Filho (Department of Health, Feira de Santana State University), Jane de Carlos Santana Capelli (Federal University of Rio de Janeiro, campus Macaé), Jerusa da Mota Santana (Health Sciences Center, Federal University of Recôncavo da Bahia), José Guilherme Cecatti (Department of Obstetrics and Gynecology, School of Medical Sciences, University of Campinas), Juliana dos Santos Vaz (Postgraduate Program in Nutrition and Foods, Faculty of Nutrition, Federal University of Pelotas), Juraci Almeida Cesar (Postgraduate Program in Public Health, Federal University of Rio Grande), Katrini Guidolini Martinelli (Postgraduate Program in Collective Health, Federal University of Espírito Santo), Lívia Castro Crivellenti (Postgraduate Program in Public Health, Department of Social Medicine, Ribeirao Preto Medical School, University of São Paulo), Luana Patrícia Marmitt (Postgraduate Program in Bioscience and Health, University of Western Santa Catarina), Marco Fabio Mastroeni (Postgraduate Program in Health and Environment, University of Joinville Region), Maria Angélica A Nunes (Postgraduate Studies Program in Epidemiology, School of Medicine, Federal University of Rio Grande do Sul), Maria Antonieta de Barros Leite Carvalhaes (Botucatu Medical School, São Paulo State University), Maria do Carmo Leal (Department of Epidemiology and Quantitative Methods in Health, National School of Public Health, Oswaldo Cruz Foundation), Maria Inês Schmidt (Postgraduate Studies Program in Epidemiology, School of Medicine, Federal University of Rio Grande do Sul), Maria Fernanda Larcher de Almeida (Federal University of Rio de Janeiro, campus Macaé), Mayra Pacheco Fernandes (Postgraduate program in Epidemiology, Department of Social Medicine, Federal University of Pelotas), Michael Eduardo Reichenheim (Department of Epidemiology, Institute of Social Medicine, Rio de Janeiro State University), Michele Drehmer (Postgraduate Studies Program in Epidemiology, School of Medicine, Federal University of Rio Grande do Sul; Postgraduate Studies Program in Food, Nutrition and Health, Department of Nutrition, School of Medicine, Federal University of Rio Grande do Sul), Mônica Araujo Batalha (Nutritional Epidemiology Observatory, Department of Social and Applied Nutrition, Josue de Castro Nutrition Institute, Federal University of Rio de Janeiro), Nathalia Cristina de Freitas-Costa (Nutritional Epidemiology Observatory, Department of Social and Applied Nutrition, Josue de Castro Nutrition Institute, Federal University of Rio de Janeiro), Patricia de Carvalho Padilha (Department of Nutrition and Dietetics, Josué de Castro Nutrition Institute, Federal University of Rio de Janeiro), Renato Passini Junior (Department of Obstetrics and Gynecology, School of Medicine, University of Campinas), Renato Teixeira Souza (Department of Obstetrics and Gynecology, School of Medicine, University of Campinas), Silmara Salete de Barros Silva Mastroeni (Health Sciences Center, University of the Joinville Region), Silvia Regina Dias Medici Saldiva (Hospital of Clinics, Faculty of Medicine, University of São Paulo), Sílvio O. M. Prietsch (Postgraduate Program in Public Health, Federal University of Rio Grande), Simone Seixas da Cruz (Department of Epidemiology, Federal University of Recôncavo da Bahia), Sirlei Siani Morais (Department of Obstetrics and Gynecology, School of Medical Sciences, University of Campinas), Sotero Serrate Mengue (Postgraduate Studies Program in Epidemiology, School of Medicine, Federal University of Rio Grande do Sul), Thaís Rangel Bousquet Carrilho (Nutritional Epidemiology Observatory, Department of Social and Applied Nutrition, Josue de Castro Nutrition Institute, Federal University of Rio de Janeiro), Vera Lúcia Bosa (Postgraduate Program in Food, Nutrition and Health, Department of Nutrition, Federal University of Rio Grande do Sul).
