# Supplementary material for: Hemoglobin Concentrations and Prevalence of Anemia During Pregnancy: Results from the Brazilian Maternal and Child Nutrition Consortium
Source: Curr Dev Nutr. 2025 May 8;9(6):107458. doi: 10.1016/j.cdnut.2025.107458 (PMC12162020; doi:10.1016/j.cdnut.2025.107458)
Supplement: multimedia component 1 [file mmc1.pdf]

Hemoglobin concentrations and prevalence of anemia during pregnancy: Results from the Brazilian Maternal and Child Nutrition Consortium

SUPPLEMENTARY MATERIAL

Nathalia Cristina de Freitas-Costa, Thais Rangel Bousquet Carrilho, Paula Normando da Costa, Helena Mendes Constante, Elizabeth Fujimori, Ana Paula Sayuri Sato, Gilberto Kac, on behalf of the Brazilian Maternal and Child Nutrition Consortium.

TABLE OF CONTENTS

Supplemental Table 1. Distribution of the concentrations of hemoglobin (g/dL) and the prevalence of anemia according to maternal characteristics removing Hb data collected through capillary samples (n= 17,565). 2

Supplemental Figure 1. Median of maternal hemoglobin during pregnancy according to the method used to calculate gestational age. 4

Supplemental Figure 2. Distribution of hemoglobin during pregnancy according to the method used to calculate gestational age. 5

Supplemental Figure 3. Distribution of hemoglobin during pregnancy with values identified as implausible considering the z score of the Intergrowth-21st curves and the z score of the sample distribution by trimester. 6

Supplemental Figure 4. Heterogeneity assessment of hemoglobin during pregnancy in the studies participating in the Brazilian Maternal and Child Nutrition Consortium. 7

Supplemental Figure 5. Distribution of hemoglobin (g/dL) during pregnancy according to the percentiles of data distribution and the Intergrowth-21st charts – sensitivity analysis removing Hb data collected through capillary samples. 8

Supplemental Figure 6. Distribution of maternal hemoglobin (A) and prevalence and 95% confidence intervals for anemia (B) during pregnancy according to the year of the data collection – sensitivity analysis removing Hb data collected through capillary samples. 9

Supplemental Figure 7. Distribution of maternal hemoglobin (A) and prevalence and 95% confidence intervals for anemia (B) during pregnancy according to the gestational trimester and maternal age – sensitivity analysis removing Hb data collected through capillary samples. .. 10

Supplemental Table 1. Distribution of the concentrations of hemoglobin (g/dL) and the prevalence of anemia according to maternal characteristics removing Hb data collected through capillary samples (n= 17,565).

Variables	Distribution of hemoglobin		Prevalence of anemia	
	Median (IQR)	Range (Min-Max)	% (n)	95% CI
Overall	12.0 (11.2-12.8)	7.4-16	13.9 (2441)	12.5-15.3
Maternal age (years) (n=11,885)				
15 – 19	11.8 (11.0-12.6)	7.4–16.0	17.4 (540) [abc]	16.0-18.7
20 – 24	12.0 (11.2-12.7)	7.5–15.8	14.8 (738) [ad]	13.8-15.7
25 – 34	12.1 (11.3-12.9)	7.7–16.0	12.2 (935) [bd]	11.4-12.9
35 – 49	12.1 (11.3-12.9)	7.5–15.4	12.8 (228) [c]	11.3-14.4
Maternal education (schooling years) (n=11,841)				
0 – 4	11.9 (11.1-12.7)	7.7–15.7	15.9 (243) [ab]	14.0-17.7
5 – 8	11.9 (11.1-12.7)	7.4–16.0	16.7 (865) [cd]	15.7-17.7
9 – 11	12.1 (11.3-12.9)	7.7–16.0	12.9 (1046) [ace]	12.2-13.7
12 – 18	12.2 (11.5-12.9)	7.7–16.0	10.2 (275) [bde]	9.0-11.3
Pre-pregnancy BMI* (n=6244)				
Underweight	11.9 (11.0-12.7)	8.8–14.8	18.6 (97) [ab]	15.3-22.0
Normal	12.0 (11.2-12.7)	7.5–16.0	15.1 (865) [cd]	14.2-16.1
Overweight	12.1 (11.3-12.9)	7.9–15.4	11.9 (249) [ac]	10.5-13.3
Obesity	12.2 (11.4-13.0)	7.5–15.8	9.2 (93) [bd]	7.5-11.0
Gestational trimester (n= 11,885)				
First (4-13 weeks)	12.6 (11.9-13.2)	8.4–16.0	6.6 (379) [ab]	6.0-7.2
Second (14-29 weeks)	11.8 (11.1-12.5)	7.5–16.0	11.2 (643) [ac]	10.4-12.0
Third (27-43 weeks)	11.7 (11.0-12.4)	7.4–15.9	23.4 (1419) [bc]	22.3-24.4
Macroregion (n=11,885)				
North	12.0 (11.1-12.7)	7.7–16.0	15.2 (185) [a]	13.1-17.2
Northeast	12.0 (11.2-12.8)	7.9–16.0	14.9 (476) [b]	13.6-16.1
Southeast	12.0 (11.2-12.8)	7.4–16.0	14.0 (1249) [c]	13.3-14.8
South	12.0 (11.2-12.8)	7.8–16.0	13.8 (450) [d]	12.6-15.0
Mid-West	12.3 (11.6-13.2)	8.1–15.7	8.2 (81) [abcd]	6.5-9.9

Notes: CI: confidence interval; [a,,b, c, d and e]. Upper script letters represent differences between the variables categories using lack of overlapping 95% CI. Anemia was defined according to WHO threshold: Hb < 11, <10.5, and <11 g/dL at 1st, 2nd, and 3rd trimesters, respectively (World Health Organization 2024). BMI: Body mass index. **BMI was classified differently for adolescents and adults, according to WHO. For adults (age > 19 years), BMI (kg/m^2) was classified as underweight (< 18.5), normal weight (≥ 18.5 and < 25.0), overweight (≥ 25.0 and < 30.0), and obesity (≥ 30) (WHO Expert Committee on Physical Status 1995). For adolescents (age ≤ 19 years), BMI-for-age z-scores were calculated considering the women's age according to WHO thresholds (World Health

Organization 2006) and classified according to the cutoffs proposed for the Brazilian Food and Nutrition Surveillance System.

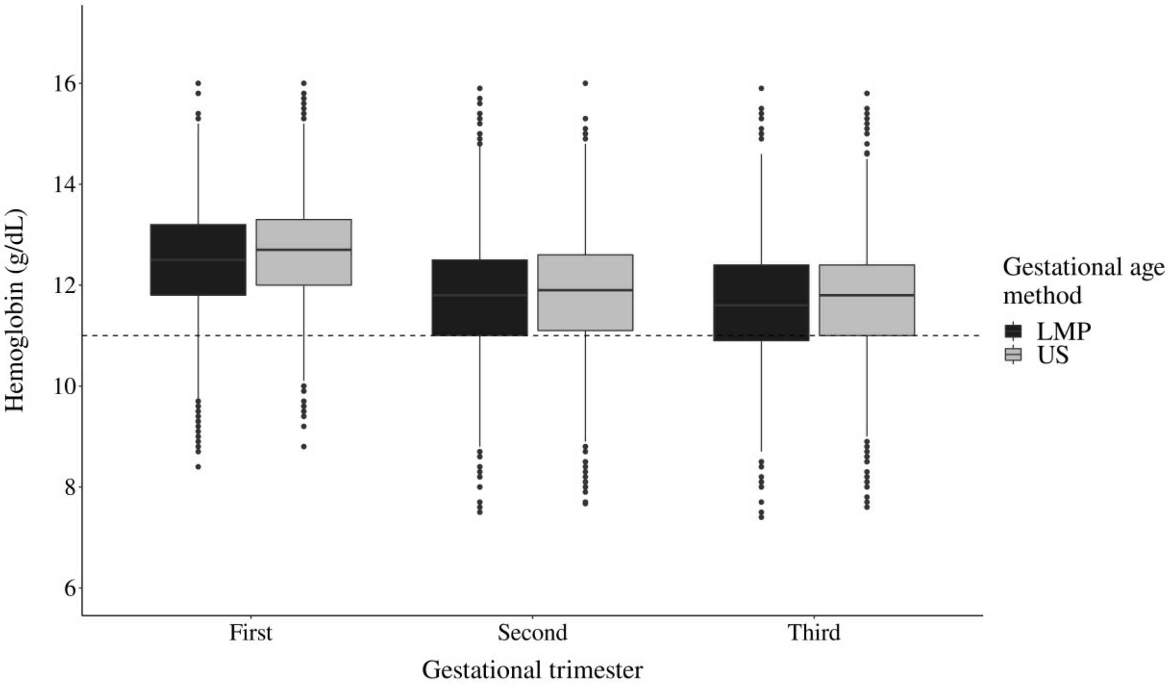

Supplemental Figure 1. Median of maternal hemoglobin during pregnancy according to the method used to calculate gestational age.

Note: LMP: last menstrual period; US: ultrasound.

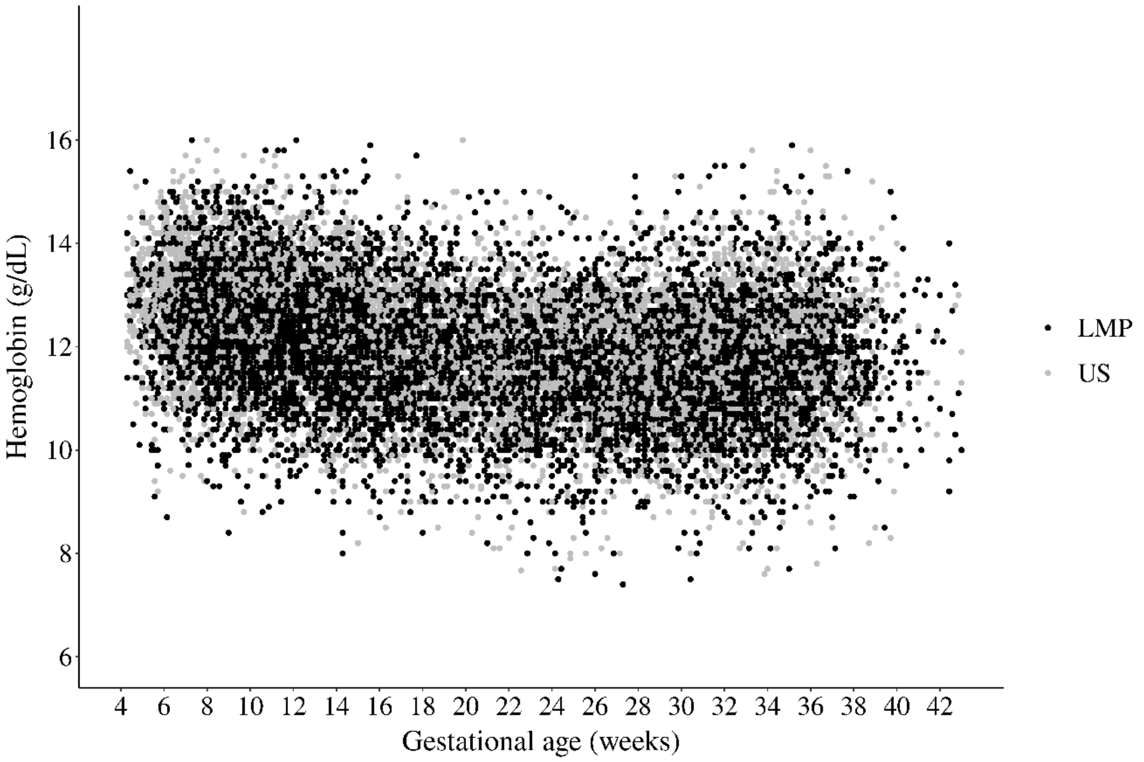

Supplemental Figure 2. Distribution of hemoglobin during pregnancy according to the method used to calculate gestational age.

Note: LMP: last menstrual period; US: ultrasound.

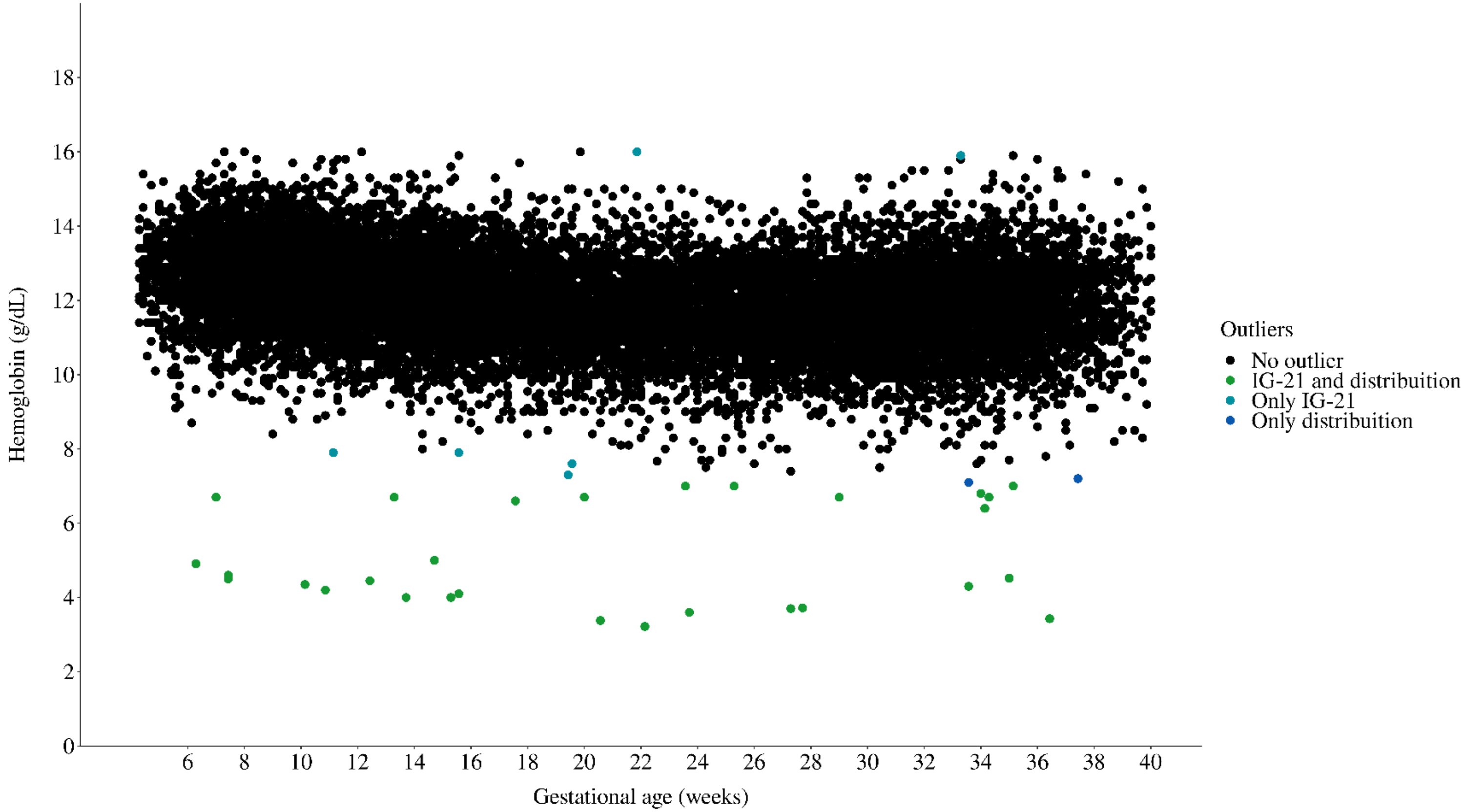

Supplemental Figure 3. Distribution of hemoglobin during pregnancy with values identified as implausible considering the z score of the Intergrowth-21st curves and the z score of the sample distribution by trimester.

Note: The outliers were identified considering the z scores of Hb according to gestational age using the Intergrowth-21st Hb standards (Ohuma et al. 2020), and values < -4 and > +4 z scores were identified as implausible.

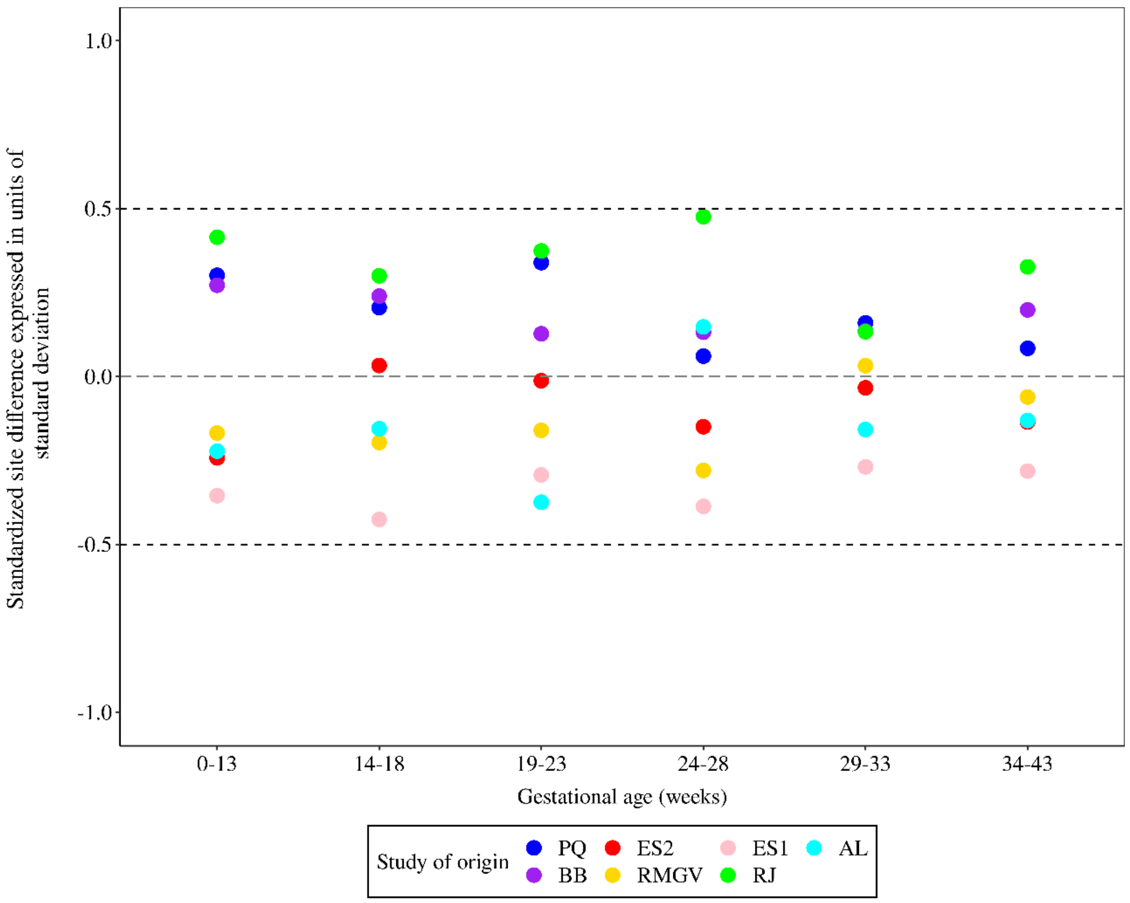

Supplemental Figure 4. Heterogeneity assessment of hemoglobin during pregnancy in the studies participating in the Brazilian Maternal and Child Nutrition Consortium.

Notes: Names of studies are derivated from acronyms and abbreviations from Portuguese: AL: Alagoas - Ferreira, 2020; BB: Birth in Brazil (Nascer no Brasil) – Leal et al., 2012; ES1: Espírito Santo 1 – Martinelli et al., 2014; ES2: Espírito Santo 2 – Polgliani et al., 2014; PQ: Petrópolis e Queimados – Marano et al., 2012; RJ: Rio de Janeiro – Farias et al., 2013; RMGV: Região Metropolitana da Grande Vitória - Santos-Neto et al., 2012.

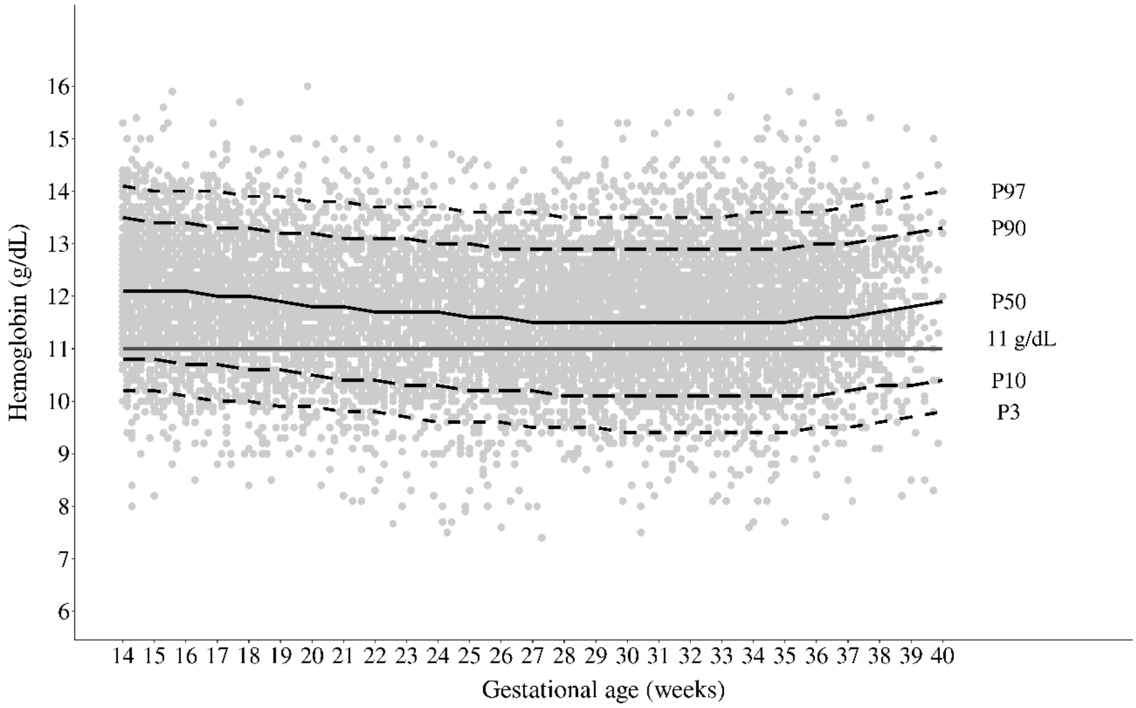

Supplemental Figure 5. Distribution of hemoglobin (g/dL) during pregnancy according to the percentiles of data distribution and the Intergrowth-21st charts – sensitivity analysis removing Hb data collected through capillary samples.

Note: Line at 11 g/dL refers to the cutoff for diagnosing anemia in 1st and 3rd gestational trimester according to the WHO. In the 2nd gestational trimester, the cutoff for diagnosing anemia is 10.5 g/dL (World Health Organization 2024).

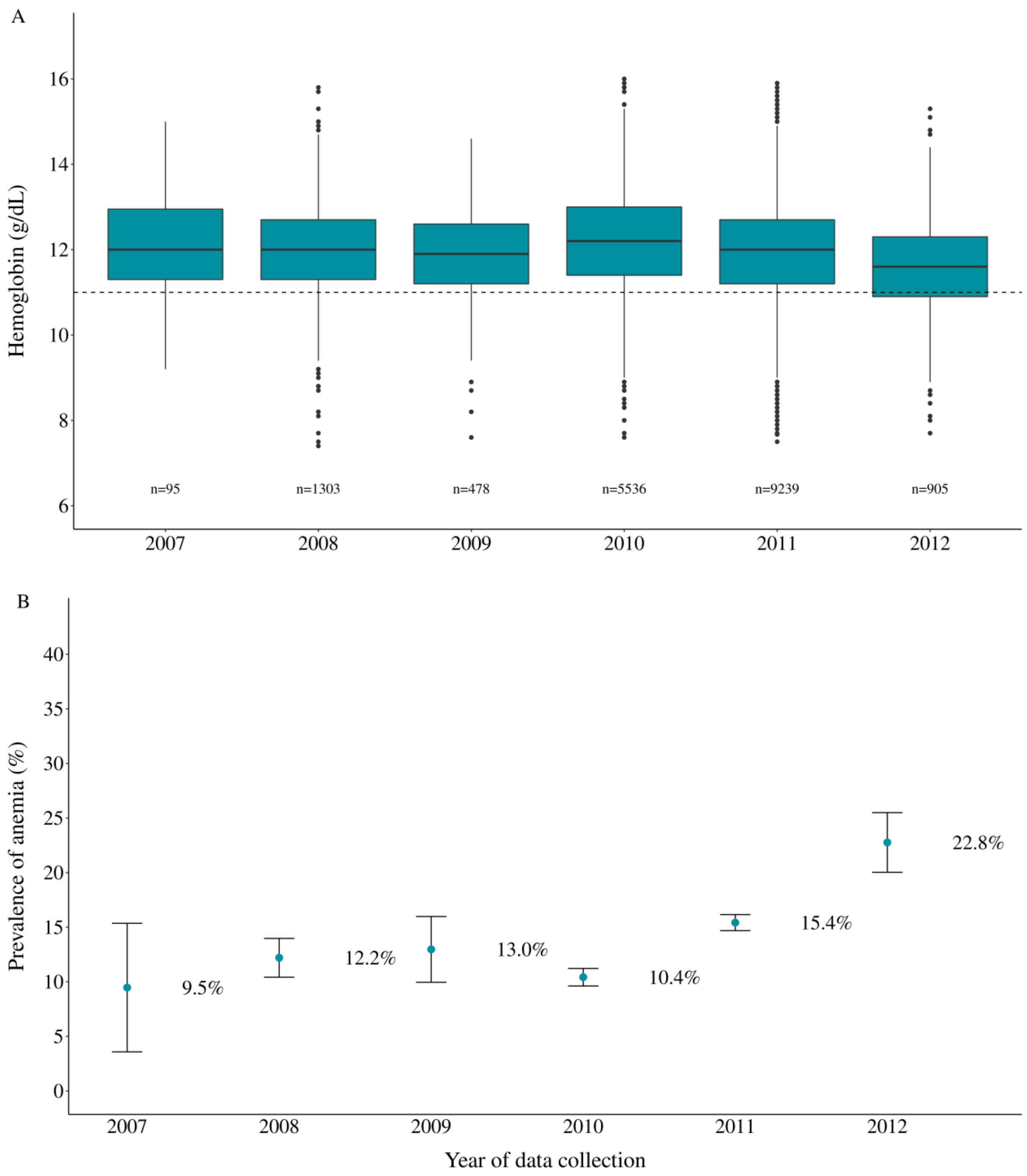

Supplemental Figure 6. Distribution of maternal hemoglobin (A) and prevalence and 95% confidence intervals for anemia (B) during pregnancy according to the year of the data collection – sensitivity analysis removing Hb data collected through capillary samples.

Note: Anemia was defined according to WHO threshold: Hb < 11, <10.5, and <11 g/dL at 1st, 2nd, and 3rd trimesters, respectively (World Health Organization 2024).

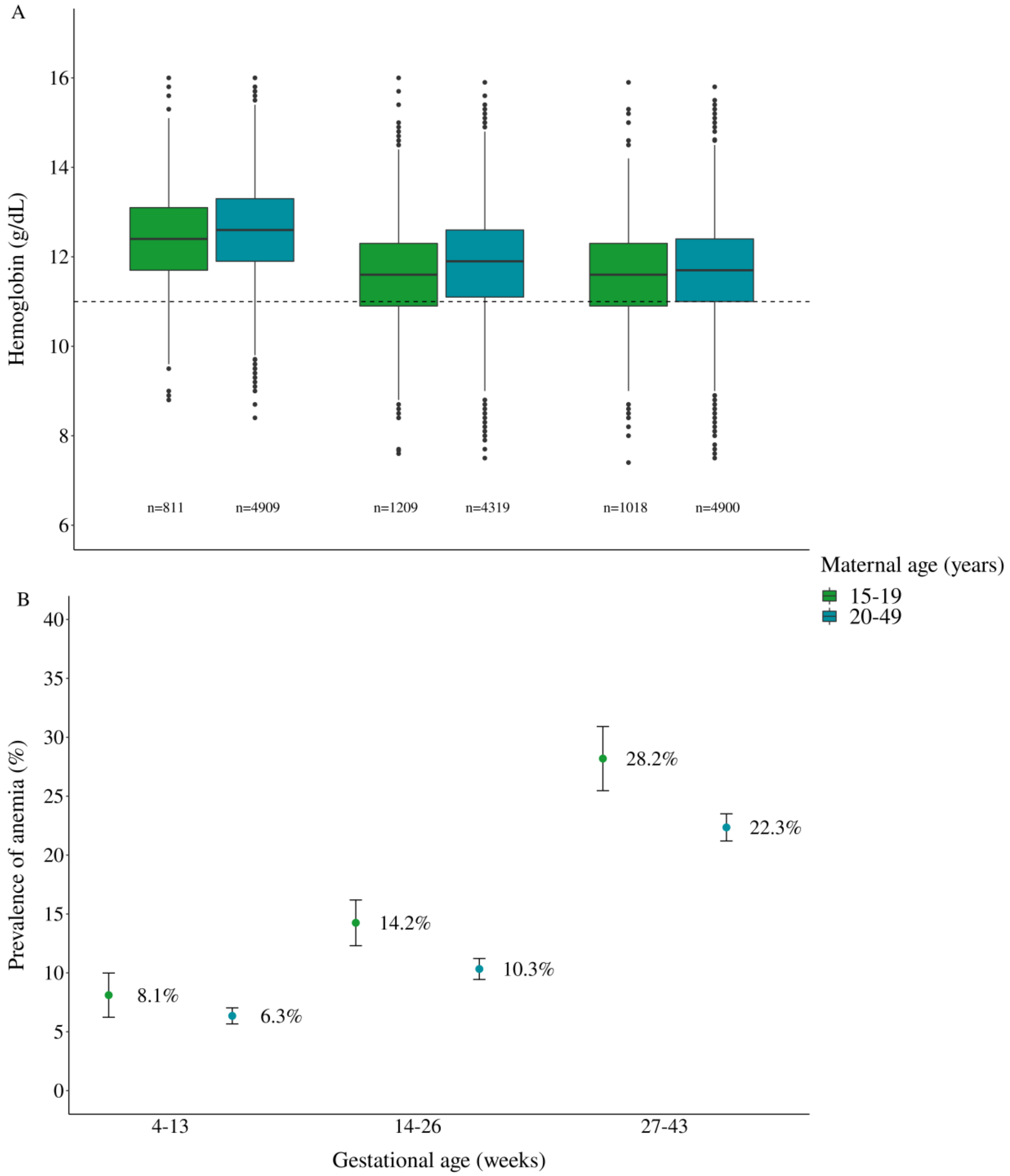

Supplemental Figure 7. Distribution of maternal hemoglobin (A) and prevalence and 95% confidence intervals for anemia (B) during pregnancy according to the gestational trimester and maternal age – sensitivity analysis removing Hb data collected through capillary samples.

Note: Anemia was defined according to WHO threshold: Hb < 11, <10.5, and <11 g/dL at 1st, 2nd, and 3rd trimesters, respectively (World Health Organization 2024).

References

Ohuma, E. O., M. F. Young, R. Martorell, L. C. Ismail, J. P. Peña-Rosas, M. Purwar, M. N. Garcia-Casal, M. G. Gravett, M. de Onis, Q. Wu, M. Carvalho, Y. A. Jaffer, A. Lambert, E. Bertino, A. T. Papageorghiou, F. C. Barros, Z. A. Bhutta, S. H. Kennedy, and J. Villar. 2020. 'International values for haemoglobin distributions in healthy pregnant women', *EClinicalMedicine*, 29-30: 100660.

WHO Expert Committee on Physical Status. 1995. "Physical status : the use of and interpretation of anthropometry , report of a WHO expert committee." In. Geneva: World Health Organization.

World Health Organization. 2006. "WHO child growth standards : length/height-for-age, weight-for-age, weight-for-length, weight -for-height and body mass index-for-age : methods and development." In. Geneva: World Health Organization.

World Health Organization. 2024. *Guideline on haemoglobin cutoffs to define anaemia in individuals and populations* (World Health Organization: Geneva).
